# Consequences of Severe Iodine Deficiency in Pregnancy: Evidence in Humans

**DOI:** 10.3389/fendo.2020.00409

**Published:** 2020-06-19

**Authors:** Freddy J. K. Toloza, Hooman Motahari, Spyridoula Maraka

**Affiliations:** ^1^Division of Endocrinology and Metabolism, University of Arkansas for Medical Sciences, Little Rock, AR, United States; ^2^Division of Endocrinology, Diabetes, Metabolism and Nutrition, Department of Medicine, Mayo Clinic, Knowledge and Evaluation Research Unit in Endocrinology (KER-Endo), Rochester, MN, United States; ^3^Central Arkansas Veterans Healthcare System, Little Rock, AR, United States

**Keywords:** iodine, pregnancy, deficiency, severe, thyroid

## Abstract

Iodine is a necessary micronutrient for the production of thyroid hormones and normal human development. Despite the significant worldwide strategies for the prevention and control of iodine deficiency, it is still a prevalent public health issue, especially in pregnant women. Severe iodine deficiency during pregnancy and neonatal period is associated with many major and irreversible adverse effects, including an increased risk of pregnancy loss and infant mortality, neonatal hypothyroidism, cretinism, and neuropsychomotor retardation. We will review the impact of severe iodine deficiency on maternofetal, neonatal, and offspring outcomes. We will also discuss its epidemiology, classification of iodine deficiency severity, and current recommendations to prevent iodine deficiency in childbearing age and pregnant women.

## Introduction

Dietary iodine is an essential micronutrient for thyroid hormone synthesis, and adequate thyroid hormone production is required for normal human development (1). Consequently, sustained deficiency in iodine intake results in a reduction of thyroid hormone production and action, and herein significant adverse health effects, especially in pregnant women and their offspring (2). Additionally, it has been described that iodine has a central role in post-natal development and plasticity of neural tissues (3). As a result, iodine deficiency can affect fetal programming through imprinting of central nervous system cells and then exert postnatal effects (4). These adverse effects are driven through the role of thyroid hormones [thyroxine (T4) and tri-iodothyronine (T3)] in neurological development, as they are required for normal neuronal migration, myelination, and synaptic transmission and plasticity during fetal and early post-natal life (5, 6). Therefore, iodine deficiency during pregnancy has been noted as the most significant preventable cause of brain damage since the beginning of the nineteenth century and enormous global strategies to eradicate this public health problem have been issued (7).

Severe iodine deficiency (SID) during pregnancy and neonatal period is associated with many other major and irreversible adverse effects, including an increased risk of pregnancy loss and infant mortality, neonatal hypothyroidism, cretinism, and neuropsychomotor retardation (8, 9). Currently, despite the significant worldwide strategies for the prevention and control of iodine deficiency, it is still a prevalent public health issue, especially in pregnant women (10–12). In this article, we provide an overview of the epidemiology of SID during pregnancy and its consequences on maternofetal, neonatal, and offspring outcomes.

## Classification of Iodine Deficiency Severity

The median urinary iodine concentration (UIC) is the recommended test by the World Health Organization (WHO) for assessing iodine intake status in populations. Because >90% of iodine intake is excreted in the urine, UIC is an excellent measure of recent iodine intake (7, 13). When the median UIC of school-age children is <20 μg/L, a population is considered to have SID, and when it has values of 20–99 μg/L, a population is considered mildly to moderately iodine deficient (14). As there is a limited number of studies assessing the iodine status in pregnant women, the median UIC of school-age children has been comparatively used, although it may not be representative of the iodine status of pregnant women in the same regions (15). This discrepancy could be due to differences in body size, urinary volumes, renal function (glomerular filtrate rate), and iodine requirements during pregnancy (9, 16).

In 2007, an updated epidemiological criterion for pregnant women's iodine nutrition status was established by consensus/committee decision of a Technical Consultation of the WHO (17). This new criterion recommended a population median UIC cut-off of <150 μg/L for defining iodine insufficiency in any trimester of pregnancy (7). This cut-off is 50 μg/L above the median UIC threshold in school-age children and adults, reflecting the higher iodine demand and intake recommendations in pregnancy (18). However, there are still no proposed cut-offs for the severity of iodine deficiency in pregnancy (7). Thus, for research purposes usually it is assumed that the severity of iodine deficiency in pregnant women is defined similarly to children and it is corresponding to the severity of iodine deficiency observed in children and adults living in the same region (19).

## Epidemiological Aspects

Historically iodine deficiency was thought to be more likely found in mountainous regions and areas exposed to frequent flooding (20). However, available data worldwide over the past two decades show that iodine deficiency is not restricted to those areas, and has been reported in different places around the world, including industrialized countries (21). As a result, universal strategies have been recommended to mitigate this global health issue. In the early nineties, the World Health Assembly set up the goal of eliminating iodine deficiency worldwide, and the UNICEF-WHO joint committee identified and recommended the strategy of universal salt iodization to prevent iodine deficiency disorders (22).

While complete eradication of iodine deficiency has not yet been reached, there has been a recognizable reduction in iodine-deficient areas, and according to recent data, more than 70% of households consume sufficient amount of iodized salt (7, 23). Nonetheless, iodine deficiency is still a prevalent problem, particularly during pregnancy. The most recent data of the US National Health and Nutrition Examination Survey (NHANES) 2005–2010 showed that the median UIC of pregnant participants in the US was 129 μg/L, suggesting an insufficient iodine status (24). In Europe, according to a 2015 estimate, women in approximately two-thirds of countries with available data were iodine deficient during pregnancy (25). In 2017, the Iodine Global Network published the Global Scorecard of Iodine Nutrition, which reported as insufficient the iodine intakes of pregnant women from more than half of the included countries (26).

Additionally, a recent systematic review of 13 studies from different regions of the world, including 14,042 pregnant women showed that the prevalence of insufficient iodine status ranged from 16.1 to 84.0%, and the median of iodine intake was insufficient in 75% of the studies (20). However, as there is no classification for mild, moderate, or severe iodine deficiency in pregnant women, it is difficult to portray the severity of this problem. Table 1 summarizes the available evidence regarding the epidemiology of SID in pregnant and childbearing age women (10–12, 27–34) using the severity classification for the general population.

**Table 1:** Epidemiology of severe iodine deficiency in pregnant and childbearing age women.

**First author**	**Year**	**Country**	***n***	**Population**	**Median UIC (μg/L)**	**SID cutoff (μg/L)**	**Patients with SID (%)**
Dillon and Milliez (27)	2000	Senegal	4,980	Childbearing age women (Pregnant women: 462)	43.0	<20	22.7
Konrade et al. (10)	2015	Latvia	829	Pregnant women	80.8 [IQR 46.1–130.6]^*^	<20^*^	4.5
Kurtoglu et al. (28)	2004	Turkey	70	Pregnant women	30.2	<20	33.0
Markhus et al. (11)	2018	Norway	851	Pregnant women	78.0 [IQR 46.0–130.0]	<20	7.3
Mills et al. (29)	2018	USA	501	Childbearing age women planning a pregnancy	112.8 [IQR 53.6–216.9]	<20	1.7
Ovadia et al. (30)	2017	Israel	1,074	Pregnant women	61.0 [IQR 36.0–97.0]	<30	29.0
Robinson et al. (31)	2018	United Kingdom	654	Childbearing age women planning a pregnancy	108.4 [IQR 62.2–167.8]	<20	2.5
Sekitani et al. (32)	2013	Ukraine	148	Pregnant women	13.0^§^	<25	50.0
Simpong et al. (12)	2016	Ghana	120	Pregnant women	NA	<20	11.7
Stinca et al. (33)	2017	Morocco	640	Childbearing age women: 156 Pregnant women: 245 Lactating women: 239	41.0 [IQR 29.0–63.0] 35.0 [IQR 19.0–62.0] 32.0 [IQR 17.0–58.0]	<20 NA <20	8.0 NA 28.0
Wang et al. (34)	2009	China	1,751	Pregnant women: 487 Lactating women: 1,264	172.2 159.2	<20	2.5 3.4

*Not available (NA), Interquartile range (IQR), Urinary iodine concentration (UIC), Severe iodine deficiency (SID)*.

* *Creatinine-standardized urinary iodine concentration, § Mean value*.

## Maternofetal Consequences

During pregnancy, there is an increase in iodine demand and thyroid hormone production due to several factors, including increased human chorionic gonadotropin level, higher levels of circulating estrogens that result in a progressive increase in thyroxine-binding globulin levels, presence of type 2 and type 3 deiodinases in the placenta, and increased maternal renal iodine excretion (1, 35, 36). Therefore, adequate iodine intake in pregnancy is crucial for maternal health and optimal fetal development. Since the beginning of the embryonic stage to mid-gestation (20 weeks), the fetus is entirely dependent on the maternal thyroid hormones and iodine supply (5). Maternal T4 is converted through placental deiodination into T3, and T3 acts in peripheral tissues playing an important role in fetal neurodevelopment (5). Specifically, T3 binds to its receptor in cells of the central nervous system and activates the transcription and expression of genes involved in axonal and dendrite outgrowth, cell migration, synapse formation and myelination (4, 37, 38). Thus, fetal development is dependent on adequate thyroid hormone, and SID is associated with serious and irreversible fetal and offspring damages.

Moreover, SID is associated with adverse pregnancy outcomes, including miscarriage, stillbirth, neonatal mortality, and growth retardation (Table 2). The first report of the association between iodine deficiency and stillbirth/abortion was published by Kemp in 1,939 (42). Another study, including 4,980 women of West Africa, reported that among women with SID, there was an increased risk of reproductive failure (stillbirths or recurrent miscarriages) [OR = 3.64; 95%CI 2.92–4.55] compared to the group with iodine sufficiency (27). However, this study has important limitations, such as self-reported past medical history and adverse pregnancy outcomes. In 1975, Pharoah et al. (43) conducted a randomized clinical trial (RCT) in the highlands of New Guinea, which found that iodine supplementation during pregnancy decreased the incidence of stillbirths and infant mortality.

**Table 2:** Studies investigating the effect of severe iodine deficiency on pregnancy outcomes.

**First author**	**Year**	**Country**	***n***	**Design**	**Median UIC (μg/L)**	**Outcomes assessed**
Alvarez-Pedrerol et al. (39)	2009	Spain	657	Prospective study conducted on pregnant women. The association between thyroid hormones and UIC during the first and third trimesters, and birth weight was studied.	1st trimester: 95.0 3rd trimester: 104.0	1st trimester Iodine sufficiency (UIC 150–249 μg/L) vs. SID (UIC <50 μg/L) - Small for gestational age: OR 1.34 (95%CI 0.16–12.06) 1st trimester Mild iodine deficiency (UIC 100–149 μg/L) vs. SID (UIC <50 μg/L) - Small for gestational age: OR 0.37 (95%CI 0.03–5.36) 3rd trimester Iodine sufficiency (UIC 150–249 μg/L) vs. SID (UIC <50 μg/L) - Small for gestational age: OR 0.37 (95%CI 0.10–1.40) 3rd trimester Mild iodine deficiency (UIC 100–149 μg/L) vs. SID (UIC <50 μg/L) - Small for gestational age: OR 0.15 (95%CI 0.03–0.76)^*^
Dillon and Milliez (27)	2000	Senegal	4,980	Epidemiological survey on iodine deficiency disorders carried out in 1996-1997. Childbearing age women were questioned about their obstetric histories, including the numbers of miscarriages and stillbirths. Urine samples were collected, the iodine level measured.	43.0	SID (UIC <20 μg/L) vs. Iodine sufficiency (UIC 101–150 μg/L) - Reproductive failure: OR 3.64 (95%CI 2.92–4.55)^*^
Mills et al. (29)	2018	USA	501	Population-based prospective cohort study, monitored women who had discontinued contraception within 2 months to become pregnant; 329 became pregnant; had UIC measured on samples collected at enrollment, and were followed up to determine pregnancy outcomes.	112.8	SID (UIC <50 μg/L) vs. Iodine sufficiency (UIC 150–249 μg/L) - Pregnancy loss: HR 0.69 (95%CI 0.32–1.50)
Torlinska et al. (40)	2018	United Kingdom	3,140	Prospective study using data from the Avon Longitudinal Study of Parents and Children (ALSPAC). This study assessed whether iodine deficiency during pregnancy was associated with pregnancy/infant loss, or with other adverse pregnancy outcomes.	61.0	SID (UIC <50 μg/L) vs. Iodine sufficiency (UIC 150–249 μg/L) - Hypertensive disorders of pregnancy: OR 1.2 (95%CI 0.7–1.9) - Preeclampsia: OR 0.6 (95%CI 0.1–3.1) - Non-proteinuric gestational hypertension: OR 1.2 (95%CI 0.7–2.1) - Any glucose derangement: OR 1.1 (95%CI 0.5–2.3) - Gestational diabetes: OR 1.6 (95%CI 0.2–17.1) - Glycosuria: OR 0.7 (95%CI 0.2–2.0) - Hyperglycemia during pregnancy: OR 1.0 (95%CI 0.5–2.4) - Anemia during pregnancy: OR 1.2 (95%CI 0.5–2.5) - Anemia <14 days postpartum: OR 0.6 (95%CI 0.3–1.1) - Postpartum hemorrhage: OR 1.2 (95%CI 0.6–2.3) - Preterm delivery: OR 1.3 (95%CI 0.6–3.0) - Cesarean section: OR 0.6 (95%CI 0.3–1.3) - Assisted/breech delivery: OR 1.2 (95%CI 0.7–2.3) - Small for gestational age: OR 1.5 (95%CI 0.7–3.1) - Large for gestational age: OR 0.7 (95%CI 0.2–1.8)
Yang (41)	2018	China	2,347	Prospective study conducted on pregnant women. Urinary samples tested for iodine, serum samples tested for thyroid function, and questionnaires about demographic information were collected. Pregnancy outcomes were recorded and compared between different urinary iodine concentration and thyroid function groups.	203.8	Iodine sufficiency (UIC 150–249 μg/L) vs. SID (UIC <50 μg/L) - Cesarean delivery: OR 0.78 (95%CI 0.50–1.21) - Preterm delivery: OR 1.38 (95%CI 0.32–6.01) - Gestational diabetes: OR 0.32 (95%CI 0.03–3.23) - Preeclampsia: OR 0.12 (95%CI 0.01–0.87)^*^ - Gestational hypertension: OR 0.49 (95%CI 0.13–1.89) - Placenta previa: OR 0.06 (95%CI 0.01–0.69)^*^ - Abnormal amniotic fluid: OR 0.51 (95%CI 0.22–1.20) - Fetal distress: OR 0.10 (95%CI 0.02–0.64)^*^ - Umbilical cord entanglement: OR 0.55 (95%CI 0.22–1.38) - Low birth weight: OR 3.73 (95%CI 0.69–20.28) - Macrosomia: OR 1.19 (95%CI 0.53–2.69) - Small for gestational age: OR 0.89 (95%CI 0.45–1.78)

*Urinary iodine concentration (UIC), Severe iodine deficiency (SID), Odds ratio (OR), Hazard ratio (HR), Confidence interval (CI)*.

* *Statistically significant difference between groups*.

Similarly, a prospective cohort study published in 2018 showed that among 2,347 pregnant women, participants with iodine sufficiency (UIC 150–249 μg/L) had decreased rates of preeclampsia [adjusted OR = 0.12; 95%CI 0.01–0.87], placenta previa [adjusted OR = 0.06; 95%CI 0.01–0.69], and fetal distress [adjusted OR = 0.10; 95%CI 0.02–0.64] compared with participants with SID (UIC <50 μg/L) (41). In a Hispanic population (*n* = 239), pregnant women with mild iodine deficiency had a lower risk of having a small for gestational age newborn compared with those women with UIC <50 μg/L [adjusted OR = 0.15; 95%CI 0.03–0.76] (39). Nevertheless, it is necessary to consider specific limitations of these studies, including relatively small sample sizes with inadequate power to assess differences in rare outcomes, samples' collection at variables gestational ages, and lack of consideration of thyroid autoimmunity as a critical factor in pregnancy outcomes. Finally, treating pregnant women with oral iodized oil just before conception or during the first trimester significantly increased placental and birth weights in a region of endemic goiter area in Algeria (44). The same study reported that in the treated group the rates of abortion (0%), prematurity (10.8%) and stillbirth (9.0%) were significantly lower than in the untreated group (19.0, 14.3, and 20.4%, respectively) (*p* < 0.001 for all) (44).

In contrast to these findings, some recent evidence has shown that there is no association of SID with some adverse maternofetal outcomes. In a population-based cohort study including 329 pregnant women, the risk of pregnancy loss was not elevated in the severely deficient group [defined as UIC <50 μg/L] (HR=0.69; 95%CI 0.32–1.50) compared to the iodine sufficient group (29). Torlinska et al. (40) used data from 3,140 singleton pregnancies of the Avon Longitudinal Study of Parents and Children (ALSPAC) to assess whether iodine deficiency during pregnancy was associated with adverse pregnancy outcomes. They reported that the incidence of pre-eclampsia, non-proteinuric gestational hypertension, gestational diabetes, glycosuria, anemia, postpartum hemorrhage, being small for gestational age, mode of delivery, preterm delivery, and large for gestational age was not significantly different among severely iodine deficient pregnant women (UIC/Creat <50 μg/g) compared to the iodine sufficient group (40). However, given the low number of adverse pregnancy outcomes in these studies, it is possible that they were underpowered to detect the effects of SID during pregnancy. In addition, it may suggest that different confounding factors associated with SID could be at play in influencing obstetric outcomes in different settings (40).

Finally, another frequently attributed impact of SID before and during pregnancy is the induction of maternal hypothyroxinemia (6, 45). Although there is no direct evidence supporting the role of iodine deficiency as a cause of maternal or fetal hypothyroxinemia in humans, this has been suggested by a few clinical studies showing that iodine supplementation during pregnancy reduces the risk of maternal hypothyroxinemia (46–48). Hypothyroxinemia is defined by free T4 levels significantly below those considered ideal for a given gestational age. In such cases, the low free T4 levels lead to increased thyroidal stimulation via the pituitary feedback mechanisms (TSH secretion) and, ultimately to goiter formation in both the mother and fetus. In areas of SID, increased rates of goiter and thyroid nodules during pregnancy have been reported (49–51) and the supplementation of iodine has been associated with reduction of maternal thyroid size (52).

## Neonatal and Offspring Consequences

SID and its treatment also affect neonatal/infant anthropometric parameters, thyroid function, nutritional status, and mortality. In a severely iodine-deficient area of western China, iodine supplementation in 295 pregnant women increased head circumference and reduced the rate of microcephaly from 27 to 11% (*p* = 0.006) (53). Treatment with iodized oil before conception or during pregnancy resulted in higher birth weights compared with the offspring of untreated mothers in Zaire (+3.7%) (54) and Algeria (+6.3%) (44). In a large Asian study, the use of iodized salt during pregnancy correlated with increased weight for age and an increased mid-upper-arm circumference during infancy (55). However, it is possible that pregnant women with adequate iodine intake had a better socioeconomic and nutritional status, which might have resulted in better anthropometric indices in their offspring. Ultrasonographic measurements of thyroid volume in neonates showed that thyroid sizes were 40% larger in the newborns from non-supplemented mothers compared with newborns from iodine-supplemented mothers. Moreover, glandular hyperplasia was already present in 10% of these neonates, compared with none in the group whose mothers received iodine (45, 56).

Neonatal and infant survival rates improved in infants born to women whose iodine deficiency was corrected before or during pregnancy. Delong et al. (57) added potassium iodate to irrigation water in three areas with SID in China and found a significant reduction in neonatal and infant mortality in these areas after 2–3 years compared with areas that did not receive iodine. In Zaire, the supplementation of iodized oil intramuscularly to severely iodine deficient pregnant women at 28 weeks of gestation significantly decreased infant mortality rate compared to the untreated group (113/1000 vs. 243/1000) (58). In a large cross-sectional study, the use of adequately iodized salt in a severely deficient area in Indonesia was associated with a significantly lower prevalence of child malnutrition and mortality in neonates, infants, and children aged <5 years (59, 60). Nevertheless, the findings of this study carry a risk of misclassification, as the adequacy of iodized salt was assessed with a rapid test, and its sensitivity and specificity were not tested against the iodometric titration method (gold standard). Another trial from Pharoah et al. (61) evaluated the effect of a single intramuscular injection of either iodized oil or saline solution on neonatal mortality. They showed that neonatal mortality rates were lower in the iodized oil-injected group (13.3%) than in the untreated group (18.2%).

When severe enough, iodine deficiency induces fetal hypothyroxinemia. Any degree of thyroid hormone unavailability during critical periods of brain development may produce irreversible brain damage, and consequently, neurocognitive delay and neurological abnormalities of the offspring (5, 45, 61). The extent of these consequences depends upon the timing and severity of the hypothyroxinemia. The most severe manifestation of *in utero* iodine deficiency is cretinism, which has been described in two forms: neurological and myxedematous (hypothyroid) (62). Approximately 4%−10% of pregnant women who are severely iodine deficient give birth to a child with cretinism (54, 58, 63, 64). Neurological cretinism is the most common form, and its clinical features include neurocognitive delay, hearing and speech defects (deaf-muteness of varying degree), squint, impaired voluntary motor activity (spastic diplegia or paresis of the lower limbs), and stance disorders (spastic gait and ataxia) (62, 65, 66). Myxedematous cretinism is characterized by severe or long-standing hypothyroidism with the following features: myxedema, dwarfism, delayed maturation of body parts, skeletal retardation, dry, thickened skin, sparseness of hair and nails, sexual retardation, deep hoarse voice, delayed tendon reflexes, and poor bowel function (62, 65, 66). Although some degree of goiter might be expected in children with myxedematous cretinism, conversely, thyroid atrophy appears to be a common finding (65). Despite the fact that these types of cretinism have distinct clinical manifestations, it has been described that both types share some neurological features, including neurocognitive delay, pyramidal/extrapyramidal dysfunction, deafness, and primitive reflexes (65, 66). Therefore, the two forms of cretinism might be part of a spectrum of diffuse insult to the developing fetal nervous system, where post-natal thyroid hormone deficiency, in the case of myxedematous cretinism, may result in their characteristic manifestations (65, 66).

The evidence linking SID to cretinism comes from landmark trials of iodine repletion during pregnancy in areas with SID in Papua New Guinea (61) and Zaire (54). In these trials, iodine supplementation using iodized oil was associated with a significant reduction in the prevalence of endemic cretinism at 4 years [RR = 0.27; 95%CI 0.12–0.60] and at 10 years of age [RR = 0.17; 95%CI 0.05, 0.58] (53) and with higher psychomotor development scores at 72 months in the offspring of iodine-treated mothers (91 ± 13 vs. 82 ± 14 in the controls) (54). Cao et al. (53) published a study in an area of SID and endemic cretinism in western China, including groups of children from birth to 3 years (*n* = 689) and women at each trimester of pregnancy (*n* = 295) (53). The intervention was oral iodized oil, and participants were followed for 2 years. The prevalence of moderate/severe neurological abnormalities among the offspring of those mothers who received iodine in the first or second trimester was 2%, compared with 9% among the infants whose mothers received iodine during the third trimester or after birth. The prevalence of microcephaly was higher in the untreated group compared with the treated group, and the mean developmental quotient at 2 years of age was higher in the treated than in the untreated children (90 ± 14 vs. 75 ± 18) (53). In a follow-up study of the same population, school-age children of the women treated before the end of their second trimester achieved higher psychomotor scores than those from mothers treated in the third trimester (86.2 vs. 81.5; *p* = 0.02), but no differences in psychological or language development tests were found (67). However, this study used tests that may not have been entirely appropriate for a population of children of that age. These data suggested that iodine supplementation before the beginning of the third trimester might reduce the negative influences of SID on the nervous system, while later intervention could not reverse the impairment of neurological status.

Trials in Peru and Ecuador also demonstrated benefits in the rates of cretinism and neurodevelopment with the use of iodized oil during pregnancy (63, 64). In Peru, women of childbearing age from Andean villages in an area of SID received either iodized oil injection or nothing, and cognitive development scores of their offspring were assessed between 1 and 4 years of age (63). Initially, there was no statistical difference in cognitive outcomes between groups; however, when children's iodine status was evaluated, there was a significantly higher intelligence quotient (IQ) score in the iodine-sufficient group compared with the iodine-deficient group (85.6 ± 13.9 vs. 74.4 ± 4.8) (63). Fierro-Benitez et al. (64) studied two villages with severe intellectual disability and cretinism rates in Ecuador. One village received an injection of iodized oil, whereas the other was not treated. Both villages were followed at 4-year intervals for 20 years, and the neurocognitive effects on the offspring were recorded. Mean IQ measured in first and second grade children was higher in the treated village than in the control village. Five years after the beginning of the trial, the treated group was stratified according to the period when iodine was initiated. The group of children whose mothers had received iodine before conception had significantly higher IQ than the group of children born after treatment had begun or the group whose mothers received iodine during pregnancy (76.8 vs. 72.3 vs. 65.2, respectively) (64). However, it is relevant to highlight that this study evaluated only the school performance of children enrolled in elementary school, who had completed at least 1 year of education. Therefore, these findings do not reflect the effect of inadequate iodine intake during pregnancy on the group of children that were not enrolled in elementary school or quitted during their first year of education.

In 2005, a meta-analysis aimed to summarize the evidence regarding the effects of iodine on the intellectual development of Chinese children showed a profound intelligence deficit in children exposed to SID, with a loss of 12.45 IQ points. With an adequate iodine supplementation for mothers before and during gestation, the IQ of their children was increased by 8.7 points (68). Additionally, studies in the Chinese population several decades ago showed that people born and continuing to live in regions of iodine deficiency, even if it is moderate deficiency, had impaired intellectual and neuromotor manifestations across a broad spectrum of severity (60–62, 65, 69, 70). Thus, this evidence highlights the importance of iodine supplementation during the neonatal period and infancy, primarily through its supplementation in breastfeeding mothers, as direct supplementation of iodine in neonates has been shown to be less effective in improving infant iodine status (71).

## Current Recommendations

As many studies have established the consequences of SID and the benefits of iodine supplementation during pregnancy, there are specific recommendations to prevent iodine deficiency in childbearing age and pregnant women. In 2007, the WHO and International Council for Control of Iodine Deficiency Disorders guidelines suggested an iodine intake of 200–300 μg/day for pregnant and lactating women (7). Similarly, the Endocrine Society guidelines (72) recommended that women of childbearing age should have an average iodine intake of 150 μg/day, and it should be increased to 250 μg/day during pregnancy and breastfeeding. The same guideline also recommends the use of once-daily prenatal vitamins containing 150–200 μg iodine before conception and during pregnancy. The most recent guidelines issued by the American Thyroid Association (73) suggested that all pregnant women should ingest ~250 μg iodine daily, and women who are planning pregnancy or are currently pregnant, should add to their diet a daily oral supplement that contains 150 μg of iodine. The above guideline also recommends that in low-resource countries and regions where salt iodization or daily iodine supplements are not feasible, a single annual dose of 400 mg of iodized oil for pregnant women and women of childbearing age can be utilized as a temporary measure. Despite some discrepancies in these guidelines, in general, there is an agreement in the need to increase iodine intake during pregnancy, and in the use of daily iodine supplements before conception, during pregnancy, and lactation.

## Conclusion

Adequate iodine status is essential for maternal and fetal health. SID during pregnancy results in irreversible, detrimental maternofetal and offspring effects. Considering the increased requirements for iodine intake during pregnancy, supplementation, even before conception, is recommended.

## Author Contributions

FT, HM, and SM have made a substantial, direct and intellectual contribution to the work, and approved it for publication. All authors contributed to the article and approved the submitted version.

## Conflict of Interest

SM receives support by the Arkansas Biosciences Institute, the major research component of the Arkansas Tobacco Settlement Proceeds Act of 2000. This material is the result of work supported with resources and the use of facilities at the Central Arkansas Veterans Healthcare System, Little Rock, AR. The remaining authors declare that the research was conducted in the absence of any commercial or financial relationships that could be construed as a potential conflict of interest.

## Abbreviations

ALSPAC: avon longitudinal study of parents and children
CI: confidence interval
HR: hazard ratio
IQ: intelligence quotient
NHANES: National Health and Nutrition Examination Survey
OR: odds ratio
RCT: randomized clinical trial
RR: relative risk
SID: severe iodine deficiency
T3: tri-iodothyronine
T4: thyroxine
TSH: thyroid-stimulating hormone
UIC: urinary iodine concentration
US: United States
WHO: World Health Organization.
